# Behaviour Detection and Recognition of College Basketball Players Based on Multimodal Sequence Matching and Deep Neural Networks

**DOI:** 10.1155/2022/7599685

**Published:** 2022-05-24

**Authors:** Long Zhang

**Affiliations:** Department of Sports, Huanghe Jiaotong University, Jiaozuo, Henan 450000, China

## Abstract

This study fuses multimodal sequence matching with a deep neural network algorithm for college basketball player behavior detection and recognition to conduct in-depth research and analysis, analyzing the basic components of basketball technical action videos by studying the practical application of technical actions in professional games and teaching videos from self-published authors of short video platforms. The characteristics of the dataset are also analyzed through literature research related to the basketball action dataset. On the established basketball technical action dataset, combined with the SSD target detection algorithm, the video images with cropping of human motion regions to reduce the size of image frames, generating a basketball technical action dataset based on cropped frames, reduces the amount of network training and improves the efficiency of subsequent action recognition training. In this study, by analyzing the characteristics of basic camera motion, a univariate global motion model is proposed to introduce a quadratic term to accurately express the shaking transformation, while the horizontal and vertical motion are independently represented to reduce the model complexity. Comparative experimental results show that the proposed model achieves a good balance between complexity and accuracy of global motion representation. It is suitable for global motion modeling in behavior recognition applications, laying the foundation for global and local motion estimation. On this basis, the visual feature change pattern of the key area of the scene (basketball area) is combined with the group behavior recognition based on motion patterns and the success-failure classification based on key visual information to achieve basketball semantic event recognition. The experimental results at NCAA show that the fusion of global and local motion patterns can effectively improve group behavior recognition performance. The semantic event recognition algorithm combining motion patterns and video key visual information achieves the best performance.

## 1. Introduction

With the continuous development of computer vision and artificial intelligence technologies, automated video content analysis has gradually become possible. Video analysis techniques cover a wide range, including video behaviour recognition, video target detection and tracking, video segmentation, pedestrian reidentification, video set generation, etc. Among them, video behavior recognition involves the semantic object behaviour and its relationship expression in the scene, which can realize the content parsing in video scenes. It is highly compatible with the demand for intelligent video understanding and has significant research value and broad application prospects [1]. This paper is oriented to the task of behaviour recognition from the perspective of video motion pattern analysis, deeply analyzes the characteristics of motion patterns in behaviour recognition scenes, optimizes video motion information representation, strengthens the feature extraction ability of the model for motion information, thus improves the performance and computing efficiency of the behaviour algorithm, and provides a practical way for the automatic parsing and intelligent management of video data. The fine classification and evaluation of human actions based on visual data refer to the use of computer vision technology for action category prediction, temporal fine classification, and action quality scoring of human actions occurring in videos, which belongs to the scope of computer vision [2]. Action classification can be divided into the classification of action categories and localization of action occurrence times depending on the classification objectives. Action assessment refers to the quantitative scoring of the occurring human actions, which can also be regarded as the fine classification of the quality of action completion. Action classification is the basis of action evaluation, and only after determining the action category can we design evaluation algorithms to quantitatively evaluate human actions based on action characteristics [3].

Models such as those based on convolutional neural networks occupy an important position in deep learning. Convolutional neural was first applied in the field of images, and after achieving excellent performance in this field, researchers have successively proposed algorithmic models to apply to video recognition. The camera is not just a simple video acquisition device but a narrative tool that matches the behaviour of the scene and better presents the key information in the scene to the audience through specific motion patterns. It would be an important application of deep learning in sports if it could be used to recognize the technical basketball moves that appear in videos, and such practical applications hold great promise in the online basketball community [4]. For example, it can provide a reference for technical moves for professional players, analysts, or basketball coaches, and it can also assist in the judgment of referees on the game court. Deep learning techniques such as wearing sensors on human joints to extract skeletal point location information for action recognition have significant limitations, while it is easier and more efficient to recognize human actions in dynamic videos. Therefore, many researchers have studied action recognition tasks by deep learning methods by improving these classical structures of convolutional neural networks to obtain the features of specific frames in a video and interimage timing information for action recognition [5]. A series of distinctive technical actions exist in both professional advanced leagues and amateur courts. For professional athletes, coaches, basketball enthusiasts, and other people, this action can often be recognized in an instant, and these actions have a recognized designation. Generally, when basketball fans search for these basketball technical action videos, it is usually the self-publishing authors who go to dig up the material of the relevant technical action highlights to make a collection, and the users of the short video platform passively receive the push from the video portal.

A convolutional neural network is an artificial neural network based on convolutional operations that excel in image-related tasks [6]. The convolutional layer uses convolutional operations to do operations with the whole image and uses the same weight coefficients for the same feature map, which greatly reduces the number of parameters in the convolutional neural network, allowing the network structure to remain relatively simple, avoiding the overfitting problem of the network model and enhancing the generalization of the network model. Meanwhile, the pooling operation of the pooling layer can reduce the number of neurons in building the network and keep the spatial translation invariance of the input data. The convolutional neural network structure is highly extensible, deep in layers, and has good expressiveness, which provides a basis for accomplishing the task of visual human-computer interaction. In this paper, based on convolutional neural networks, we investigate and analyze visual human-computer interaction approaches on a standard library of gestures and human behaviours from both static and dynamic aspects, respectively. In the static analysis, a static gesture standard dataset with complex backgrounds is selected, and a static gesture recognition model is trained using convolutional neural networks to achieve fast and accurate recognition of static gestures. In the rotation transformation, the motion direction of the motion vector is consistent with the rotation direction of the camera, and the amplitude transformation satisfies the distribution law of the amplitude of the rotation transformation. In the shake transformation, the points in the motion field have the same direction of motion, and the motion amplitude gradually increases along the direction of camera rotation. In the dynamic analysis, we select a standard dataset of continuous human action behaviours in a complex context, establish contextual relationships between continuous frames of human action behaviours, train a dynamic human behaviour recognition model, and achieve accurate recognition of dynamic human action behaviours.

## 2. Related Works

Action classification can be divided into action classification in cut video and temporal action detection in uncut video. Both action classification and temporal action detection are very classical vision tasks in the field of computer vision, and many research teams have conducted research on these two problems so far and achieved relatively remarkable scientific results [7]. A deep learning-based target detection algorithm R-CNN is proposed, which uses convolutional neural networks for image recognition and classification tasks. Although target detection using deep learning had been attempted before, it was not until the proposal of R-CNN that target detection became feasible for practical application. The average accuracy of R-CNN on the Pascal VOC 2012 test set reached 53.3%, and the algorithm has since inspired many deep learning-based target detection algorithms. Depending on the data source, action classification methods can be further classified into RGB video-based action classification, depth image-based action classification, and skeletal data-based action classification [8]. Compared with RGB video and depth images, skeletal data is compact, smaller, and easier to extract concise motion features, so motion classification algorithms based on 3D skeletal data are faster and more efficient. The emergence of the motion capture device Microsoft Kinect in recent years has made the acquisition of skeletal data more convenient, which makes the motion classification algorithm based on skeletal data a current research hotspot [9].

A hierarchical model for the whole multiplayer scene is proposed, which describes human behaviour from multiple levels of detail, including social role relationship models [10]. A conditional random field is proposed to model interactions between roles and person-specific social descriptors, and since social roles are described by interactions between people in an event, group behaviour recognition is performed through easy-to-process variational reasoning to simultaneously reason [11, 12]. A generative model is proposed which designs a four-layer structure (appearance pose, individual behaviour, group behaviour, and scene/event) that converts the original intralayer interactions into interlayer interactions, implements interactions between multiple groups and between multiple members of the group, and aggregates this information to obtain the final group behaviour label [13].

Even though the importance of interaction relationship features is considered, the generalized consideration of interaction information of all group members lacks the portrayal of interaction relationships for key characters, which will make the interaction relationship information redundant and reduce the recognition effect. Therefore, in this paper, we propose a model based on a graph convolutional network for inference of grouped interaction relations in complex group scenes and further refusion, in which the scene members are inferred separately in two complementary information ways.

## 3. Multimodal Sequence Matching Fusion Deep Neural Network Algorithm Design

Convolutional neural networks are constructed by scientists modeled after the structure of layer-like neural networks in the animal visual cortex. The core convolutional layer operations are inspired by the layers of simple and complex cells in the neural network structure, where the complex cells located at higher orders usually have larger receptive fields than simple cells and can respond to more complex features. However, all these cells are capable of accurately detecting and recognizing the displacement and deformation of objects.

(1) $$\begin{array}{c} G_{x}\left(x,y\right)=I\left(x-1,y\right)+I\left(x+1,y-1\right). \end{array}$$

A convolutional neural network is composed of an input layer, a convolutional layer, an activation function, a pooling layer, a fully connected layer, a loss function, and an output layer. In image processing, the input layer is usually a single frame or multiple frames, and there is usually more than one convolutional layer and pooling layer, which alternate between extracting more complex features and obtaining higher-level semantic information and delivering it to the fully connected layer [14]. It needs to be based on a certain a priori regularity of the behaviour recognition scene and has high requirements for the stability of the global information in the image edge area. This process is called the feedback operation of the convolutional neural network. The feedforward operation and feedback operation are repeated to adjust and update the parameters repeatedly until the convolutional neural network converges, and the parameters of each part are determined to form the network model.

(2) $$\begin{array}{c} G_{x}\left(x,y\right)=\sqrt{G_{x}{\left(x,y\right)}^{2}-G_{y}{\left(x,y\right)}^{2}}. \end{array}$$

At present, the learning of representations of multisource data has become the mainstream development direction in the field of computer vision. Using multimodal data as input can extract information representations of different modalities so that the recognition network can learn the importance of different modal data. However, sign language videos usually contain many transition frames and silent frames, which to some extent, affect the characterization ability of sign language data. Therefore, this paper uses keyframe sequences of multimodal sign language videos as input, thus improving the utilization of key information and the performance of the model.

(3) $$\begin{array}{c} G\left(x,y,\sigma \right)=\frac{1}{2\pi \sigma^{2}}e^{x^{2}-y^{2}/2\pi \sigma^{2}}. \end{array}$$

**Figure 1:**
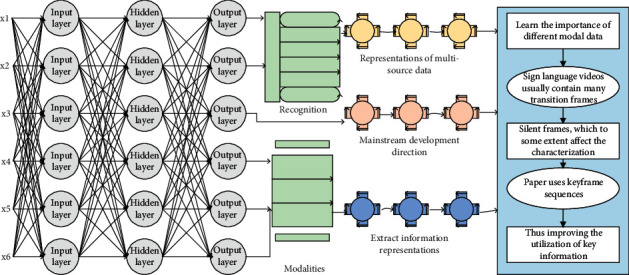
Algorithmic framework of multimodal sequence matching fusion deep neural network.

The backpropagation algorithm is mainly based on the gradient descent method to continuously update the weights among the neurons of adjacent layers of the multilayer network. Like a student learning from a modified assignment, iterative iterations are performed through incentive propagation and weight updates until the set goal of learning is reached. Weights and biases can be set at the beginning by generating random values in a Gaussian distribution. This method updates the weights of the network when the output value deviates from the set expectation, as shown in Figure 1.

Since the human brain detects simple information such as the edges of objects before combining them into a complex whole. For shallow networks to recognize high-resolution image data, the fully connected layer structure of neural networks can make the weight parameters of the model too much or even impossible to calculate. With fewer layers in the network, the weights in the model can be overtrained and lead to overfitting problems when the dataset is insufficient [15]. In such a complex situation, Euclidean distance is difficult to calculate the distance or similarity directly and effectively between two sequences. The emergence of convolutional neural networks has an important significance, and their advantages are unmatched by other models in the field of deep learning. It has many applications in processing text information, image recognition, and other fields. The process of the human eye seeing an object is as follows: pixel information enters the pupil, visual cells transmit the signal, certain cells in the cerebral cortex perceive the edge and direction of the target, and the neural network is graded layer by layer.

(4) $$\begin{array}{c} D\left(x\right)=D-\frac{\partial D^{T}}{\partial x}\Delta x-\frac{1}{2}\Delta x^{T}\frac{\partial^{2}D}{\partial x^{2}}\Delta x. \end{array}$$

The convolutional neural network is built to imitate this mechanism from vision to neural network to input, so it has a great advantage in processing a large amount of image information. Neurons in traditional neural networks are connected one to another, which leads to too much computation and thus cannot be learned. In contrast, a neuron in the convolutional layer is connected to only some neurons in the adjacent layer and not to all of them, which makes the training volume greatly reduced.

(5) $$\begin{array}{c} E_{\tau }\left(x,y,t\right)=\overset{\tau -1}{\underset{i=1}{\cup }}D\left(x,y,t+i\right). \end{array}$$

Subspace learning has the defects of high computational complexity and the inability to eliminate the correlation between orders.

(6) $$\begin{array}{c} G=\max\sum_{p=1}^{M}\left\|X_{p}^{T}U_{p}+y\right\|+\left\|Y_{F}^{2}-\lambda_{1}\sum_{p=1}^{M}\left\|U_{p}\right\|\right\|. \end{array}$$

Global motion is the motion information generated by the camera motion during video shooting, and the global motion information is mainly focused on the background region or the stationary foreground object region in the scene. Global motion is very common in video data, reflecting the camera operator's shooting intention and skill, and there is an implicit correlation between some specific scenes and behaviours [16].

**Figure 2:**
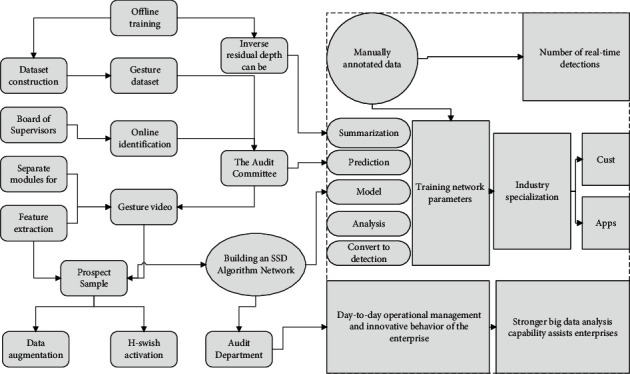
Recognition flow chart.

As shown in Figure 2, the panning transformation includes two types of up and down panning and left-right horizontal panning, which can be described as the vertical or horizontal axial rotation of the camera on a central axis, without displacement of the camera itself, to realize the transformation of the shooting scene through the rotation of the angle. Compared with the translation transformation, the camera is not level with the shooting scene plane during the panning transformation, and a rotation angle is generated between the camera plane and the original scene plane.

The camera is not just a simple video acquisition device but a narrative tool that matches the behaviour of the scene toward a specific motion pattern to better present the key information in the scene to the viewer. At the same time, the h-swish activation function shows good performance in the deep network, which further improves the recognition rate by 2.64%, but slightly reduces the recognition time by 0.005. Take a basketball broadcast video as an example. The camera will make sure the player with the ball is on the broadcast screen by panning or panning transformation after the player has finished shooting. The camera will focus on the basket area by zooming transformation so that the audience's attention is always focused on the key areas of the court, thus giving the audience a better viewing experience.

The function of zoom transformation is to switch between detailed information and macro information. Rotation transformation is one of the basic image transformations, but it is relatively rare in the behaviour recognition scene. The camera rotation is mostly caused by the irregular shaking of the shooting pivot point. The function of panning transformation is like that of panning transformation, but there is a big difference between them in the presentation and the global motion field amplitude distribution.

The figure shows the basic global motion in the behaviour recognition scene, including translation, scaling, rotation, and panning transformations, and the motion trend of points in the scene is represented by regions [17].

The establishment of the global motion model has greater relevance to the camera imaging process. The essence of camera imaging is the process of image coordinate system and view plane transformation. In the video shooting process, the real 3D dynamic scene is transformed into a digital image sequence through 2D mapping. The objects in the scene undergo a mapping process from the world coordinate system in the original scene to the camera coordinate system and are finally transformed into the pixel coordinate system in the image.

## 4. Design of Behaviour Detection and Recognition System for High School Basketball Players

The skeletal data consists of multiple joint point position coordinates, which are related to the reference coordinate system and usually differ in various real scenes. Even in the same shooting scenario, the joint point coordinates of people with the same pose may differ due to differences in height and size, sensor angle, and distance from the sensor [18]. Therefore, the original skeletal sequences need to be normalized to reduce the effects of skeletal scale and shooting angle.

**Figure 3:**
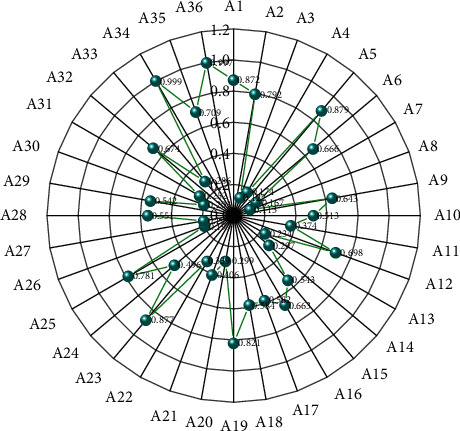
Human behaviour in MSR database.

To reduce the sensitivity of the skeletal sequence to the shooting perspective, the original skeletal sequence needs to be transformed to a reference coordinate system determined by the skeletal sequence itself. Overall, after downsampling, the average time for progressive action detection on a test video sample containing an average of 6608 frames in the DFMAD-70 database is 10.95 s. As mentioned earlier, the skeletal frame-based coordinate transformation treats each frame separately and loses the relative motion information between frames. The skeletal sequence-based transformation preserves the interframe relative motion of the original skeletal data by performing the same coordinate transformation on all skeletal frames in the sequence, as shown in Figure 3.

To verify the importance of timing information in human body behaviour, the behaviour that is in the opposite order of the original depth map sequence is referred to as the inverse order behaviour in this paper. The inverse order behaviour in this paper is obtained by arranging the depth map sequence of the positive order behaviour in reverse order operation to obtain the new databases D1, D2, where D1 is the MSR database and the MSR inverse order database, and D2 is the UTD database and the UTD inverse order database.

The advantage of the global motion estimation algorithm based on statistical analysis is that the algorithm is fast, the complexity of the algorithm is low, and the data dependence is small. However, the robustness of the algorithm has certain shortcomings, and it needs to be built on the certain a priori regularity of the behaviour recognition scene, which has high requirements for the stability of global information in the image edge region. Among them, video behaviour recognition involves semantic object behaviour in the scene and its relationship expression, which can realize the content analysis in the video scene, which is highly compatible with the needs of intelligent video understanding and has great research value and broad application prospects. When there is a large proportion of local motion points in the edge region. There is an error in the estimation of the motion field in the edge region, and the algorithm will produce a biased estimation and affect the accuracy of the global motion estimation results.

**Figure 4:**
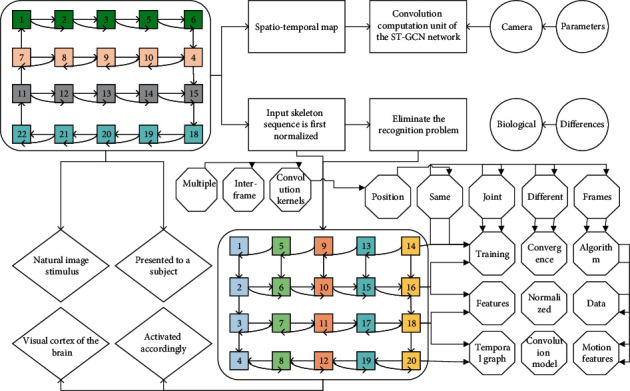
Multipolygon subspace learning.

The TEM module is added to each layer of the Spatiotemporal map convolution computation unit of the ST-GCN network, and the input skeleton sequence is first normalized to eliminate the recognition problem caused by different camera parameters and biological differences. The joint position of the same joint varies greatly in different frames during training, and the convergence of the algorithm will be affected if the position features are not normalized. The normalized data are fed into the time-domain extended Spatiotemporal graph convolution model, and the motion features of the joint points within frames are firstly extracted with multiple convolution kernels, then the temporal features of the adjacent joint points are extracted with multiple interframe convolution kernels in the time-domain extended graph convolution module, and finally, the motion features are extracted in the long time dimension using the traditional time-domain graph convolution. After the multilayer time-domain expanded Spatiotemporal graph convolution unit, the 256-dimensional feature vector of each sequence is obtained by aggregating the node features with the average pooling layer, which represents the features of the whole skeleton graph, and then the features are classified with the SoftMax function to calculate the probability of which class of action the sequence belongs to, and the one with the highest probability is generally selected as the judgment class of the action, as shown in Figure 4.

Nonglobal motion points can affect the performance of global motion estimation algorithms, which mostly use iterative optimization to gradually remove the estimation bias caused by local motion. However, neural network models are robust to noise components and can generalize the essential feature expressions from the data to suppress the noise components.

In the test phase, a mixed motion field is an input, and all the raw data in its horizontal component and all the column data in its vertical component are formed into a batch input to the network, and the corresponding global motion estimation results are output [19]. Improving the performance and computing efficiency of behavioural algorithms provides a feasible way for automatic analysis and intelligent management of video data. To further mitigate the impact of local motion noise components on the global motion estimation process, an optimization scheme based on statistical information of the data is adopted in this paper. Taking the horizontal motion field as an example, since the output result ideal state column data should have the same value, we use a statistical analysis strategy to sort the column data and take the middle 30% data of the sorted result to calculate the mean value as the uniform result of this column data.

## 5. Experimental Design for Motion Detection and Recognition

In the field of signal processing, time series are a common representation of data. A common task of time series processing is to compare the similarity between two series. Usually, the similarity measure of a time series requires the calculation of distance information between series. A more direct, simple, and common distance measure is the direct calculation of the Euclidean distance between the input template and the reference template. The calculation of the Euclidean distance is only suitable for measuring the similarity of two equal-length time series. Human behaviour is highly random, and the length of video sequences describing behaviour often varies due to different actors or different time points of occurrence; for example, in the case of walking, some people prefer to walk fast while others prefer to walk slowly. In such a complex situation, it is difficult to calculate the distance or similarity between two sequences directly and effectively by Euclidean distance.

**Figure 5:**
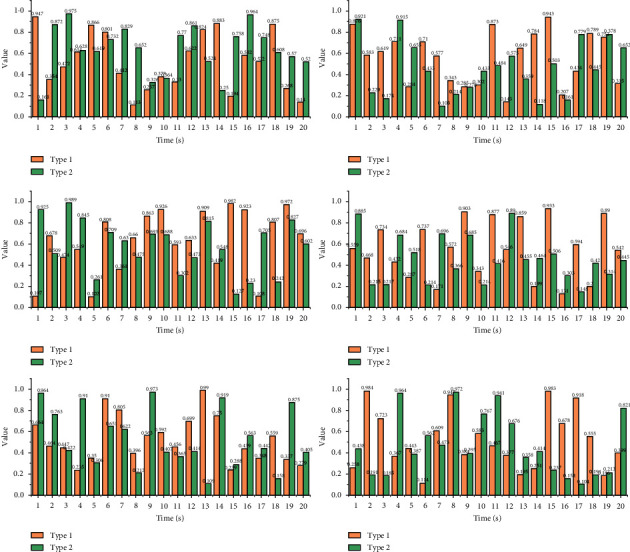
Euclidean distance between two-time series.

As shown in Figure 5, the top and bottom lines, respectively, represent the two-time series. The waveforms of the two sequences have similar trends, but they cannot be aligned on the time axis. Like the characteristics possessed by two different time series of the same type of behaviour, the similarity of the two-time series is measured using Euclidean distance by aligning the two series directly vertically on the time axis and calculating the distance between the eigenvalues at the corresponding time points. Such a calculation method cannot effectively measure the similarity between two-time series. Therefore, time regularization of the preprocessed data is necessary before the similarity analysis of the behavioural sequences.

The behavioural pattern recognition model based on the DTW algorithm usually contains a data preprocessing module, feature extraction module, template set training module, and matching analysis module. The main function of the data preprocessing module is to optimize the original data and increase the number of training samples by normalization, angle rotation, coordinate translation, etc. If the basketball technical movements that appear in the video can be recognized, this will be an important application of deep learning in sports. The main function of the feature extraction module is to extract the behavioural features in the video frame sequences and transfer them to the template set for parameter training. The template set training module gets the matching parameters of the temporal structure algorithm by learning the feature parameters.

The classification recognition of human behaviour patterns in video sequences is completed by matching analysis. The algorithm solves the problem of temporal uncertainty of human behaviour better, even if the test sequence and the reference sequence do not have the same duration. If the temporal order constraint is satisfied, the template matching between the sequences can be completed better. The algorithm has the advantages of computational simplicity and robustness. However, the computational efficiency decreases significantly with the increase of the number of samples, and therefore, the algorithm is not suitable for analyzing complex behaviour.

## 6. Analysis of the Results

### 6.1. Algorithm Performance Analysis Results

**Figure 6:**
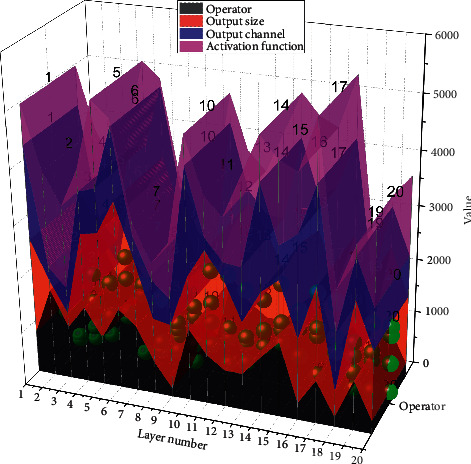
Structure of the prediction network.

The prediction network has 12 layers of the network, as shown in Figure 6, and the operators are general 2D convolution and deep convolution. The role of deep convolution is to compress the feature map size, similar to the pooling operation, which is located at layers 19, 22, 25, and 28. The size of the candidate frames of the feature maps for prediction is 19 × 19, 10 × 10, 5 × 5, 3 × 3, 2 × 2, and 1 × 1, where the 19 × 19 and 10 × 10 feature maps are located in layers 14 and 17 of the feature extraction network, and the remaining feature maps are located in layers 20, 23, 26, and 29 of the prediction network, respectively.

The recognition time refers to the time required to recognize a single gesture image and is usually calculated as the average recognition time, which is transformed by the number of frames (FPS) that the network model can perform gesture recognition in 1 s. When basketball enthusiasts search for these basketball technical action videos, it is usually self-media authors to dig out the materials of relevant technical action highlights to make a collection, and short video platform users passively receive the push of the video portal. And since the shorter the time required for a single gesture image, the faster the gesture recognition, the recognition time is usually considered as an evaluation metric.

**Figure 7:**
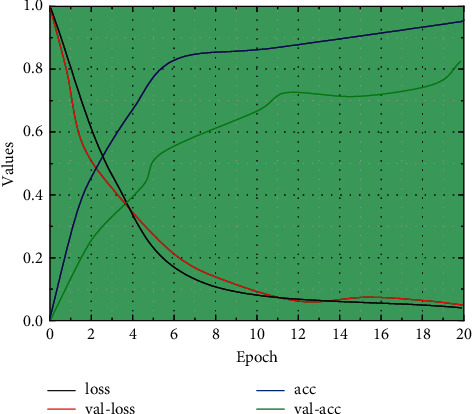
Model training trend graph.

The inverse residual depth-separable module can reduce the loss of feature information and improve the recognition rate by 0.65% than using the VGG16 network directly, and adding the depth-separable convolutional structure to it makes the average recognition time improve by 0.020 s/frame; meanwhile, the h-swish activation function shows good performance in the deep network, which makes the recognition rate further improve by 2.64%, but the recognition time is slightly reduced by 0.005 s/frame. In gesture recognition, the inverse residual depth-separable module and the h-swish activation function are introduced at a small-time cost but can improve the recognition accuracy relatively large, proving the effectiveness of the inverse residual depth-separable module and the h-swish activation function, as shown in Figure 7.

For the human behaviour recognition algorithm based on a convolutional neural network, a large amount of data is needed to support the algorithm training to ensure that the algorithm can learn the features of each type of data well. The 20 BN-jester public dataset used for training in this paper is a medium-sized dataset, which is still far from the data required by the algorithm; at the same time, there are more than 5 million frames in the dataset for training, validation, and testing, which have high requirements on the storage and computing functions of hardware devices. In the static analysis, the standard dataset of static gestures under complex background is selected, and the static gesture recognition model is trained by the convolutional neural network to realize the fast and accurate recognition of static gestures. Therefore, in this paper, human behaviour recognition uses the idea of migration learning to fine-tune the parameters of the training network. The model parameters of the homologous convolutional neural network trained in the large dataset challenge are selected as the initial parameters of the convolutional neural network in the long-term bidirectional recurrent convolutional network in this paper, which can solve the phenomenon of underlearning caused by the insufficient amount of data.

The model obtained at the 14th epoch (the lowest value-loss) is the final network model. The loss of the model at the 14th epoch is 0.2678, Val-loss is 0.9616, act is 0.9274, and Val-act is 0.7508. 77.36% of the frames in the 20 BN-jester test set are tested with the obtained human behaviour model, and some of the recognition results are shown in Figure 7.

### 6.2. Experimental Results

**Figure 8:**
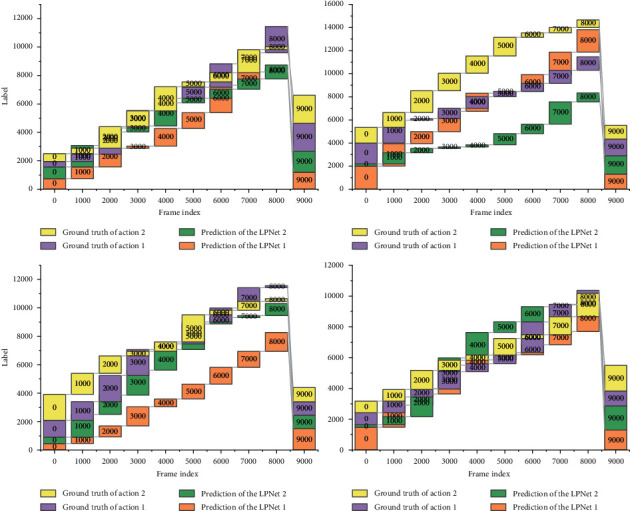
Single category response characteristics of the progress tag sequence.

The jump shot is a technique in which the offensive player suddenly stops with the force of his legs and knees while traveling, and then the defending player is out of position due to inertia in the process of rushing back to the defense, and then the offensive player shoots. The main point of the action is to adjust the hands into a shooting position between the dribble in the line, and the position of the player's hands will change significantly when turning the basketball. Skilled players are good at using this technique, adjusting their hands now of closing the ball to make the position of their hands on the basketball as close as possible to make it easier to shoot. And the best time to close the ball is the last dribbling action before breaking through the rush to collect the ball; when the offensive player completes the last dribble to collect the ball instantly, step out of the first step while driving the second step to move, through the way of broken steps to form a pad, the pace is small to move a short distance, play a role in adjusting the centre of gravity and feel. Thus faster-moving speed also makes the defender too late to react. The high-level semantic information is obtained and sent to the fully connected layer, which is called the feedforward operation of the convolutional neural network. Then through the calculation of the loss function, the parameters of each layer are updated from the last fully connected layer forward. At the same time, as the basketball rises, the offensive player usually uses one-handed contact with the basketball, through experience and familiarity with the ball sense, to quickly adjust the best hand shot position. Many post players use such a padded rush stop not only because of the flexibility of movement, but the moment of the rush stop to collect the ball can also play a role in bending the knee to build up strength for the next shot, as shown in Figure 8.

The basic visual characteristic of the Euro step is the larger left-right stride of the human body, which causes the defender to be misled due to the large left-right sway and affects his judgment of the attacking player's shot. The footwork often taps the basketball on the last dribble before the ball is closed, cleverly using the definition of the zero-step in the basketball walk rule, a technique that can also cause defenders to misjudge. The first step after the ball combined a smaller step compared to the second step, followed by a larger second stride from one side of the defender to the other to break through. The key to this action is that the stride forms a confusing action of downward abduction, substantially swinging the ball by holding it with both arms, and the inertial impact of the stride, legally using the shoulder to form a certain confrontation with the defender to create the space to strike.

Take the right-hand direction as the direction of attack. For example, when the attacking player faces the defending player, the eyes observe the defending player, through the right palm stomp, step to the left foot to the right side, the right wrist into a parallel angle to half hold the ball but avoid closing the ball to avoid causing the second transport for example. After that, the body half-turn state using centripetal force, and the left foot, with explosive force, stomps away. After shooting the ball once, switch to the left hand, pull the ball to the left side of the body and accelerate beyond the defense from the left side. The selection criterion for the turning action in this video set is the view behind the character to ensure that the turning as an action feature can be captured.

**Figure 9:**
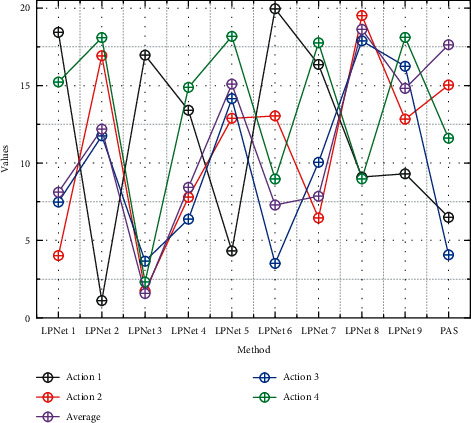
Running time of the progressive motion detection algorithm.

This section analyzes the time complexity of the proposed algorithm by giving the running efficiency of the algorithm on the DFMAD-70 database. Due to the small number of network layers, the weights in the model will be overtrained, and when the dataset is insufficient, it will also lead to overfitting. Progressive action detection for each video sample consists of two parts, first generating a sequence of progress labels using the progress label prediction network and then applying the PAS algorithm to complete the action detection and the time consumed by each part is shown in Figure 9. Among the 36 test samples in the DFMAD-70 database, the number of predicted data belonging to Action 1, Action 2, and Action 3 are 114842 frames, 65902 frames, and 57146 frames, respectively, so the average number of frames contained in the test video samples is 6608 frames, and the average time to generate the progress label sequence is 289.75 s. The time complexity of the PAS algorithm is also related to the length of the progress label. Overall, the proposed progressive motion detection algorithm takes about 306.42 s to localize the motion of one video sample from DFMAD-70 data.

It is mentioned that by downsampling the original video, not only a high mAP can be maintained, but also the time complexity can be greatly reduced. The algorithm time complexity after downsampling the video with a sampling step of 32 is also given in Figure 9. The generation of the progress label sequence is linearly related to the number of video frames, and the generation time of the progress label sequence after downsampling is about 1/26 of the original one. The PAS algorithm is more sensitive to the length of the progress label sequence, so the running time of the PAS algorithm is greatly reduced to about 1/1667 of the original one. The downsampling operation effectively improves the runtime of the PAS algorithm. Overall, after downsampling, the average time for progressive motion detection is 10.95 s for the test video samples containing 6608 frames on average in the DFMAD-70 database, which greatly improves the detection efficiency of the progressive motion detection algorithm and enables fast progressive motion detection.

## 7. Conclusion

When the algorithm model detection and recognition performance is improving, its application is worthy of expectation nowadays. Meanwhile, a short video is a product of the rapid development of the mobile Internet era. A self-media short video is given the spirit of the Internet, creating a new form of information dissemination. Basketball fans are no longer unilaterally and passively receiving information but also can participate in the creation. We find a community of like-minded people and participate in the production of content. In addition, the scale of short video users is gradually expanding, and the popularity of self-media has enriched the spiritual and cultural life of society, providing more opportunities for people to entertain and learn. Among many sports, basketball has significant technical action features. A video basketball technical action recognition algorithm based on a dual-resolution 3D-CNN architecture is constructed for action recognition after building a CNN network. The effectiveness of this paper's algorithm is verified through comparison experiments of original frames, cropped frames, and SVM classification after fusing features on the basketball technical action dataset.

## Data Availability

The data used to support the findings of this study are available from the corresponding author upon request.
